# From Waste to Green: Water-Based Extraction of Polyphenols from Onion Peel and Their Adsorption on Biochar from Grapevine Pruning Residues

**DOI:** 10.3390/antiox12091697

**Published:** 2023-08-31

**Authors:** Melissa Prelac, Igor Palčić, Danko Cvitan, Dominik Anđelini, Maja Repajić, Josip Ćurko, Tvrtko Karlo Kovačević, Smiljana Goreta Ban, Zoran Užila, Dean Ban, Nikola Major

**Affiliations:** 1 Institute of Agriculture and Tourism, Karla Huguesa 8, 52440 Poreč, Croatia; melissa@iptpo.hr (M.P.); danko@iptpo.hr (D.C.); dominik@iptpo.hr (D.A.); tvrtko@iptpo.hr (T.K.K.); smilja@iptpo.hr (S.G.B.); zoran@iptpo.hr (Z.U.); dean@iptpo.hr (D.B.); nikola@iptpo.hr (N.M.); 2 Department of Food Engineering, Faculty of Food Technology and Biotechnology, University of Zagreb, Pierottijeva 6, 10000 Zagreb, Croatia; maja.repajic@pbf.unizg.hr (M.R.); josip.curko@pbf.unizg.hr (J.Ć.)

**Keywords:** adsorption capacity, bioactive compounds, biomass valorization, green chemistry, phytochemicals

## Abstract

Onion peels (OP) are rich in bioactive compounds with a plethora of benefits for human health, but this valuable material is often wasted and underutilized due to its inedibility. Likewise, grapevine pruning residues are commonly treated as agricultural waste, but biochar (BC) obtained from this material has favorable characteristics as an adsorbent. This study investigated the potential of BC in removal of targeted polyphenolic compounds from OP extracts. The OP extracts were obtained adhering to green chemistry principles using deionized water amplified by three methods: maceration (MAC), ultrasound-assisted extraction (UAE), and microwave-assisted extraction (MAE). The extraction efficiency on the polyphenolic profile and antioxidant capacity was investigated with different extraction temperatures and solid-to-liquid (s/l) ratios. For further analysis, UAE at 90 °C with an s/l ratio of 1:100 was used due to higher polyphenolic compound yield. The BC adsorption capacity of individual polyphenols was fitted with the Langmuir and Freundlich isotherm models. Quercetin-3,4′-diglucoside obtained the highest R^2^ coefficient in both models, and the highest q_max_ value. The optimum conditions in the dosage experiment suggested an amount of 0.5 g of BC using 3 g/L extracts. The studied BC showed a high affinity for targeted phytochemicals from OP extracts, indicating its potential to be applied for the green adsorption of valuable polyphenolic compounds.

## 1. Introduction

The *Allium* genus is a part of the *Alliaceae* family, counting more than 750 species [1] with large morphological and phenotypical diversities, including wild and domesticated species, mostly distributed in the northern hemisphere [2,3]. Onion (*Allium cepa* L.) has been domesticated for over 4000 years [2], as evidenced by a Sumerian written paper dating from 2600–2100 BC that mentions onions [3] and an Egyptian onion image mural from around 3000 BC [1]. 

According to the FAO [4], more than 200 million tons of onions and shallots were produced worldwide in 2019, making *A. cepa* one of the most cultivated horticultural crops. In 2021, 7.1 million tons of onions were harvested in the EU, mostly in the Netherlands (27.1%) and Spain (20.7%) [5].

Onions are grown for their edible multilayer tissue bulb [6] and are consumed fresh, boiled, or baked, as well as processed: pickled or dehydrated as a powder, minced, or granulated [3,7]. Onions are widely used in human nutrition [1,8] due to their flavor [9], nutritional value [1,10,11], and health benefits such as cancer prevention, cardiovascular disease prevention, obesity prevention, erectile dysfunction prevention, and anti-inflammatory and hepatoprotective properties [3]. The future potential of onion cultivation is based on the development of cultivars rich in phytochemicals [3]. 

Onion is a biennial bulb crop rich in bioactive compounds, the most abundant of which are polyphenols [12]: flavonoids and flavanols, with high antioxidant activity due to their ability to scavenge free radicals from oxygen and fatty acids, and alk(en)yl cysteine sulfoxides [13,14,15]. Flavonoids are a class of plant secondary metabolites with a polyphenolic structure divided into a few subgroups as flavones, flavonols, isoflavones, flavanones, anthocyanins, and flavanols [11,16]. Onion bulbs are abundant in spiraeoside (quercetin-4′-glucoside), rutin, and quercetin in bulbs [12]. Many authors have reported the positive influence on human health when polyphenolic compounds present in onions are consumed [14,15,17]. Besides the positive effect on human health, this group of phytochemicals has many biological functions in plants [16]. 

Besides the bulb, onion peel contains high amounts of polyphenolic compounds as well, particularly flavonoids [18,19]. According to many authors [15,19], the major compounds identified in onion peel are quercetin, quercetin glucosides, and their oxidative products, as well as cyanidin-3-glucoside as an anthocyanin representative. The content of flavonoids in onion peel is recognizable by the color, which mostly varies from yellow to red or brown. Quercetin and its derivatives give a yellow-colored onion skin, while anthocyanins are responsible for the reddish-colored ones [11,18]. According to a study onion peel is 10-fold richer in total polyphenolic content compared to onion flesh [20]. Furthermore, onion peel has 99.68 mg of quercetin per gram of powder, compared with 2.35 mg of quercetin per gram of powder in onion flesh.

Although onion peel is rich in bioactive compounds, it is not edible and is not used as food [18,19,20], resulting in wastage of high-value phytochemicals. Moreover, food waste has a negative environmental impact, producing 8–10% of global greenhouse gas emissions [21], contributing to the impact of global warming [22]. As reported in Eurostat [5], approximately 57 million tons of food waste (127 kg/inhabitant) was generated in 2020 only in the EU. Many authors have reported on the possibility of reusing food waste to produce biofuel [23], biopolymers [24], chemical products [25], and compost [26]. Food waste has the potential to be used for cosmetic, pharmaceutical, or food industrial purposes due to its high bioactive compound content, wide availability, and low-cost source [18,19,27,28]. The biological properties of polyphenols are being used in the production of enriched functional foods, to increase their level of antioxidant capacity and enhance one or more biological activity aspects [29]. Recently, there has been an increased interest among many scientists and even the general population in the research and consumption of functional foods [30], and onion extracts were frequently used to enrich food products, such as chicken, minced sardines, corn oil, and turkey [17].

Agriculture, and in this context viticulture, generates a considerable amount of biomass waste [31]. Pyrolysis is a thermal degradation process, ordinarily in oxygen-limited conditions, resulting in charcoal, bio-oil, and fuel gas production [32]. Biochar is a carbon-rich, non-homogeneous, low-polar material with a porous structure, usually obtained by pyrolysis [33,34]. Biomass pyrolysis represents an alternative solution in organic waste management [35]. Many authors [34,36] have reported about the potential of biochar in environmental remediation due to its adsorption capacity. With the presence of negatively charged organic functional groups, cation exchange capacity, and a large surface area due to a large distribution of pores [37], biochar is described as an efficient adsorbent. The presence of essential functional groups such as carboxylic (-COOH), hydroxyl (-OH), amine, amide, and lactonic on the surface of biochar increase its sorption capacity [38]. Biochar adsorption capacity is a complex phenomenon affected by various factors, such as electrolyte content, temperature, pH, surfactant structure, pore volume, and the nature of the activated carbon used [39,40]. As previously reported, biochar from grapevine pruning residues is an efficient adsorbent of polyphenolic compounds [41]. 

This research aimed to evaluate water as a green extraction solvent of polyphenolics from onion peel and the suitability of biochar as an adsorbent for bioactive compounds from onion peel extract. For the extraction experiment, three extraction methods (maceration, ultrasound-assisted extraction, and microwave-assisted extraction), five solid-to-liquid (s/l) ratios, and five extraction temperature levels were investigated, and the polyphenolic profile and antioxidant capacity of the obtained extracts were determined. Subsequently, the biochar adsorption capacity for the studied polyphenolics was fitted with Langmuir and Freundlich isotherm models.

## 2. Materials and Methods

### 2.1. Plant Material

Yellow onion (*Allium cepa* L.) peels were sourced from a local restaurant (Momjan, Croatia) as a byproduct from their operation. The outer peels were air dried at 30 °C for 24 h (Memmert UF160, Schwabach, Germany), and ground to a 0.2 mm fine powder using an ultra-centrifugal mill (Retsch ZM 200, Haan, Germany). 

### 2.2. Biochar Production

Grapevine pruning residues were collected from an experimental vineyard of the cultivar “Istrian Malvasia” (*Vitis vinifera* L.) at the Institute of Agriculture and Tourism in Poreč, Croatia. The canes were pyrolyzed at a maximum temperature of 400 °C in a Kon-Tiki system as described by Prelac et al. [41]. The obtained biochar was air dried for 24 h at 30 °C (Memmert UF160, Schwabach, Germany), then ground in a mortar mill (Retsch RM 200, Haan, Germany). The powder was sieved through a test sieve to obtain a particle size of 125 to 250 µm. 

### 2.3. Experimental Setup of Water-Based Extraction of Polyphenols from Onion Peel

To assess the performance of water as a sole green extraction solvent [42], an experiment was set up as a full factorial design with five solid-to-liquid ratios of sample mass to water, five temperature levels, and three extraction techniques. The ground onion peel was weighed at five mass levels, including 1, 0.5, 0.25, 0.1, and 0.05 g of onion peel powder and each mass level was fused with 25 mL of distilled water at 20 °C to obtain s/l ratios of 1:25, 1:50, 1:100, 1:250, and 1:500, respectively. Each s/l ratio was subjected to temperature levels of 30, 45, 60, 75, and 90 °C over 30 min with a 5 min preheating step using three techniques: (a) maceration (MAC) in a heated water bath (GFL 1013, Burgwedel, Germany); (b) ultrasound-assisted extraction (UAE) in a heated ultrasonic water bath using 300 W ultrasound power (40 kHz) (MRC 250 H, Holon, Israel); (c) microwave-assisted extraction (MAE) in a microwave unit with microwave power set at 800 W (Milestone Ethos Up, Sorisole, Italy). The extracts were allowed to cool for 24 h. Subsequently, the extracts were centrifuged at 16,000× *g* for 5 min (Domel Centric 350, Železniki, Slovenia), and the supernatant was collected and filtered through a 0.22 µm syringe filter into an HPLC vial. The extracts were stored at −18 °C until further analysis.

### 2.4. Polyphenolic Compounds Analysis in Onion Peel Extracts

To identify and quantify the phenolic compounds in onion peels, chromatographic analyses were performed on a Shimadzu Nexera UPLC-PDA instrument consisting of a degassing unit (DGU-405, Shimadzu, Kyoto, Japan), an autosampler (SIL-40CX3, Shimadzu, Kyoto, Japan), a system controller (SCL-40, Shimadzu, Kyoto, Japan), a photodiode array detector (SPD-M40, Shimadzu, Kyoto, Japan), two solvent delivery units (LC -40DX3, Shimadzu, Kyoto, Japan), a column oven (CTO-40C, Shimadzu, Kyoto, Japan), and a Poroshell 120 EC-C18 2.7 µm column (2.1 × 150 mm) (Agilent, Palo Alto, CA, USA). The temperature in the column oven was set at 40 °C. The injection volume was equalized for all ratios, starting with 2.5 µL for 1:25 s/l ratios and finishing with 50 µL for 1:500 ratios. The flow rate was set at 0.4 mL/min. Gradient elution was performed as follows: 0–18 min, 98% A to 2% B; 18–20 min, 40% A to 60% B; 20–21 min, 20% A to 80% B; 21–25 min, 2% A to 98% B; and 25–30 min, 98% A to 2% B, where solvent A was water and solvent B was methanol, both containing 0.2% of acetic acid (*v*/*v*). The total run time was 30 min. Phenolic compounds were identified and quantified using calibration curves obtained with serial standards dilutions of gallic acid (y = 5247.41x + 7854.05, R^2^ = 0.9997), protocatechuic acid (y = 6707.07x − 4641.54, R^2^ = 0.9997), quercetin-3,4′-glucoside (y = 3895.77x + 722.83, R^2^ = 0.9998), vanillic acid (y = 6402.29x − 670.60, R^2^ = 0.9999), quercetin-3′-glucoside (y = 5206.39x − 803.89, R^2^ = 0.9999), quercetin-4′-glucoside (spiraeoside) (y = 8634.89x − 1219.71, R^2^ = 0.9999), quercetin (y = 7283.94x − 4286.11, R^2^ = 0.9998), and isorhamnetin (y = 9082.21x − 3525.57, R^2^ = 0.9999). Quercetin-3,7,4′-glucoside, quercetin-3,7′-glucoside, and isorhamnetin-3,4′-glucoside were identified and quantified using quercetin-3,4′-glucoside standard, while isorhamnetin-3′-glucoside and isorhamnetin-4′-glucoside were determined using quercetin-3-glucoside standard. Separation and quantification of phenolic compounds were monitored at 280 nm and 360 nm, respectively. The results were expressed in µg/g DW of onion peel.

### 2.5. Antioxidant Capacity of Onion Peel Extracts

DPPH radical scavenging activity assay was carried out according to Brand-Williams et al. [43], with slight modifications. All extracts were diluted with an appropriate amount of distilled water, 200 µL of 0.02M freshly prepared DPPH radical was mixed with 100 µL of extract, and the well-plate was kept in the dark at 25 °C. Antioxidant capacity values were read at an absorbance of 517 nm (Tecan Infinite 200 Pro M Nano+, Männedorf, Switzerland) after 30 min of reaction time, and calculated against a calibration curve of Trolox (ranging from 20 to 100 µM, y = −13.47x + 13.407; R^2^ = 0.9998). Results were expressed as µmol of Trolox equivalents/g dry weight (µmol TE/g DW).

Ferric reducing antioxidant power (FRAP) was determined according to Benzie and Strain [44], with some modifications. Briefly, all extracts were diluted with an appropriate amount of distilled water, and a volume of 100 µL of extracts was mixed with 200 µL of freshly prepared FRAP reagent. The well-plate was stored in the dark at 25 °C for 10 min. The absorbance was measured at 593 nm (Tecan Infinite 200 Pro M Nano+, Männedorf, Switzerland). Antioxidant capacity values were calculated using a calibration curve of Trolox (ranging from 20 to 100 µM; y = 6.82156x + 0.02291; R^2^ = 0.9999). Results were expressed as µmol TE/g DW.

Oxygen radical absorbance capacity (ORAC) was determined as described by Ou et al. [45], with slight modifications. All extracts were diluted with an appropriate amount of distilled water, 37.5 µL of extracts were pipetted onto a well-plate, 225 µL of a freshly prepared 4 µM fluorescein solution was added, and the reaction mixture was incubated at 37 °C for 30 min. Finally, 37.5 µL of freshly mixed AAPH was added to the incubated mixture. Excitation (485 nm) and emission (528 nm) wavelengths were measured for 120 min (Tecan Infinite 200 Pro M Nano+, Männedorf, Switzerland). Antioxidant capacity values were calculated from a calibration curve of Trolox (ranging from 4 to 20 µM; y = 0.0404x − 0.0005, R^2^ = 0.9999). Results were expressed as µmol TE/g DW.

### 2.6. Adsorption Capacity of Grapevine Pruning Residues Biochar

For further analysis, onion peel extract obtained by UAE at 90 °C and a s/l ratio of 1:100 was used. In the first experiment, the polyphenol adsorption capacity of biochar was investigated by fusing batches of 10 mg biochar with onion peel extract at concentrations ranging from 5 to 50 mg/L. In the second experiment, the effect of biochar dosage on the adsorption of polyphenolic compounds was investigated. Here, biochar at a dosage of 0.5 to 2.5 g/L was fused with onion peel extract at a concentration of 3 g/L.

The mixtures were rotated for 24 h at 25 °C (Biosan Multi RS60, Riga, Latvia). The samples were filtered through a 0.22 μm filter into an HPLC vial. The analyses were performed on an LC-ESI-QqQ (Shimadzu, Kyoto, Japan). The instrument consisted of a column oven compartment (Nexera CTO-40C), an autosampler (Nexera SIL-40CX3), two solvent delivery units (Nexera LC-40DX3), and a QqQ mass spectrometer (LCMS8045). Comparing the specific ions and retention times with the analytical standards, targeted compounds were identified. Using a column C18, 2.1 × 150 mm, 2.7 µm core-shell column (Advanced Materials Technology, Wilmington, DE, USA), 1 µL of the extract was injected. The temperature in the oven was set at 40 °C. Gradient elution of mobile phases performed as follows: 0 min to 1 min, 98% A; 1 min to 16 min, 98% A to 40% A; 16 min to 21 min, 40% A to 0% A; 21 min to 24 min, 0% A; 24 min to 25 min, 0% A to 98% A; and 25 min to 30 min, 98% A, where mobile phase A was water, and mobile phase B was methanol, both containing 0.1% acetic acid (*v*/*v*). Flow was set at 0.30 mL/min. The response surface methodology was applied as the experimental design to optimize the yield of targeted polyphenolics from onion peels. Two independent variables consisting of 5 temperatures and 5 s/l ratios were used for each method with the aim to maximize the yield of investigated compounds. The studied responses were gallic acid, protocatechuic acid, quercetin-3,4′-diglucoside, quercetin-4′-glucoside, quercetin-3-glucoside, and isorhamnetin-4-glucoside, expressed in µg/g DW.

Furthermore, to better understand the adsorption dynamics of the polyphenolic compounds onto the biochar surface, the results of the first experiment were fitted with the Langmuir and Freundlich isotherms. The Langmuir isotherm is described as follows [46]:

1/q_eL_ = 1/q_max_ + 1/(K_L_ × q_max_) × 1/γ_e_ (1)

where q_eL_ represent the amount of adsorbate concentration in the solid phase at equilibrium (mg/g), 1/q_max_ is the slope of linear equation, 1/(K_L_ × q_max_) is the y-intercept, K_L_ signifies the affinity constant (L/mg), q_max_ is the maximum monolayer adsorption capacity (mg/g), and γ_e_ is the amount of adsorbate concentration in the liquid phase at equilibrium (mg/L). The equation was plotted as 1/q_eL_ vs. 1/γ_e_, and the coefficient of determination (R^2^) was calculated. Additionally, the R_L_ factor was calculated to determine the favorability of Langmuir isotherms as described:

R_L_ = 1/(1 + K_L_ × γ_0_) (2)

where K_L_ is the affinity constant (L/mg) and γ_0_ is the initial concentration of the adsorbate (mg/L). 

The Freundlich isotherms were calculated using the equation below and plotted as log q_eF_ vs. log γ_e_. The Freundlich isotherm constant (K_F_/(mg/g) × (L/g)^n^), adsorption intensity (n), and R^2^ were calculated using the plot.

log q_eF_ = log K_F_ + 1/n × log γ_e_ (3)

### 2.7. Statistical Analysis

All experiments were performed with three repetitions. Data were statistically analyzed using Statistica 13.4 (Tibco, Inc., Palo Alto, CA, USA) by analysis of variance (ANOVA), and Tukey’s post hoc test with significant differences at *p*-value ≤ 0.05 to compare the group means values. Additionally, the graphs of temperature against s/l ratio in the obtained extracts were plotted using the Distance Weighted Least Square algorithm in Statistica 13.4 (Tibco, Inc., Palo Alto, CA, USA).

## 3. Results

### 3.1. The Effect of Different Extraction Methods, Temperature, and Solid-to-Liquid Ratio on the Polyphenolic Profile and Antioxidant Capacity of Onion Peels

The polyphenolic compounds detected in the investigated samples included 13 compounds but due to poor isotherms results, only gallic acid, protocatechuic acid, quercetin-3,4′-diglucoside, quercetin-3-glucoside, quercetin-4′-glucoside, and isorhamnetin-4-glucoside were further investigated. 

**Figure 1:**
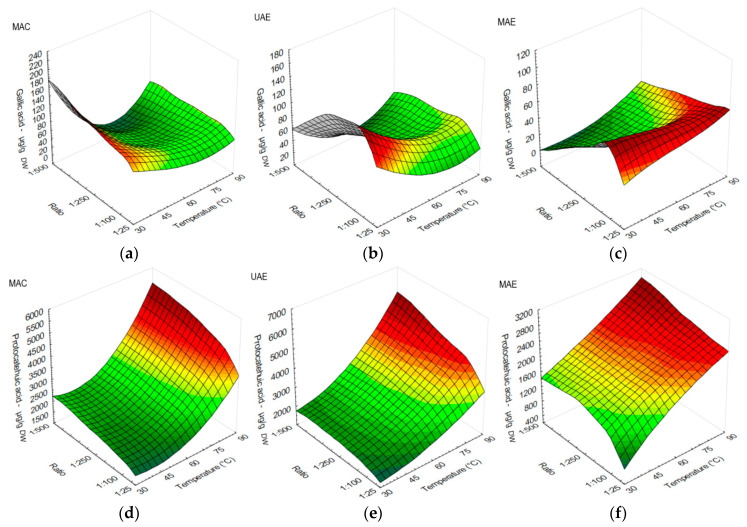
Surface plots showing the influence of temperature against s/l ratio in onion peel extracts on content of hydroxybenzoic acids: (**a**) gallic acid (µg/g DW) when using MAC; (**b**) gallic acid (µg/g DW) when using UAE; (**c**) gallic acid (µg/g DW) when using MAE; (**d**) protocatechuic acid (µg/g DW) when using MAC; (**e**) protocatechuic acid (µg/g DW) when using UAE; (**f**) protocatechuic acid (µg/g DW) when using MAE.

**Figure 2:**
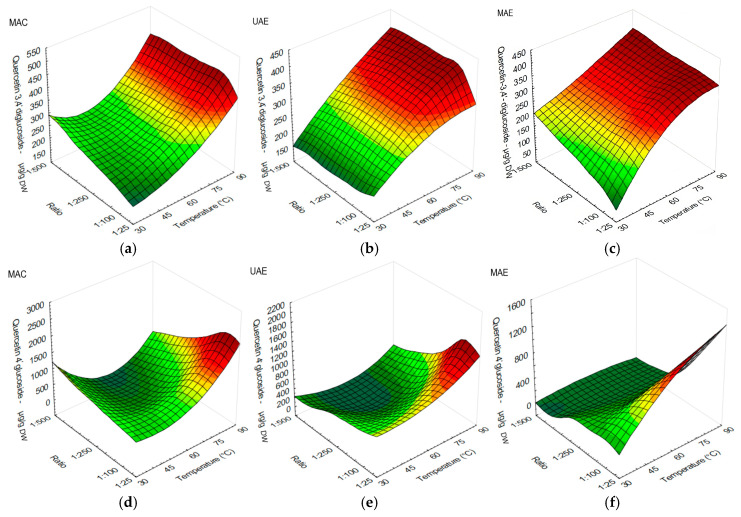
Surface plots showing the influence of temperature against s/l ratio in onion peel extracts on major flavonol and flavonoid: (**a**) quercetin-3,4′-diglucoside (µg/g DW) when using MAC; (**b**) quercetin-3,4′-diglucoside (µg/g DW) when using UAE; (**c**) quercetin-3,4′-diglucoside (µg/g DW) when using MAE; (**d**) quercetin-4′-glucoside (µg/g DW) when using MAC; (**e**) quercetin-4′-glucoside (µg/g DW) when using UAE; (**f**) quercetin-4′-glucoside (µg/g DW) when using MAE.

**Figure 3:**
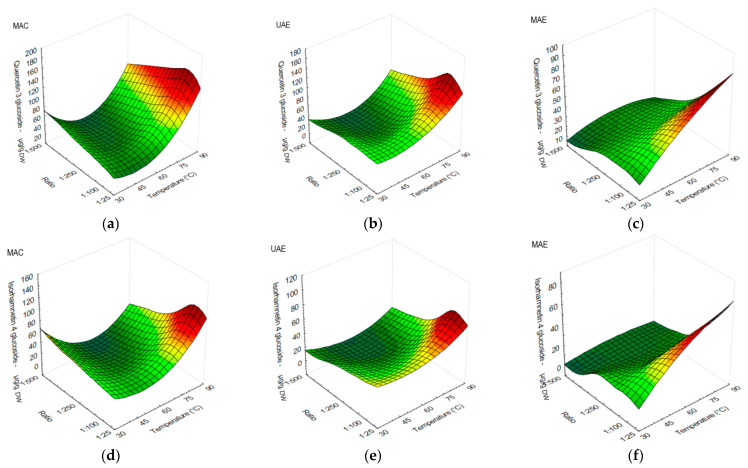
Surface plots showing the influence of temperature against s/l ratio in onion peel extracts on content of the lowest flavonoids amounts: (**a**) quercetin-3-glucoside (µg/g DW) when using MAC; (**b**) quercetin-3-glucoside (µg/g DW) when using UAE; (**c**) quercetin-3-glucoside (µg/g DW) when using MAE; (**d**) isorhamnetin-4-glucoside (µg/g DW) when using MAC; (**e**) isorhamnetin-4-glucoside (µg/g DW) when using UAE; (**f**) isorhamnetin-4-glucoside (µg/g DW) when using MAE.

The effects of temperature against s/l ratio on polyphenolic profiles using MAC, UAE, and MAE are shown in Figure 1, Figure 2 and Figure 3. All investigated compounds yielded better at 75 °C or above, with the exception of gallic acid. Gallic acid yield results were comparable when MAC and UAE were applied. Higher yields of these two applied extractions were obtained at extraction temperatures of 30–45 °C, while s/l ratio effect was not visible. The highest yields of gallic acid when using MAC were obtained at 30 °C using s/l ratio 1:500 and 45 °C using s/l ratio 1:50 (Figure 1a). Gallic acid yielded better at 30 and 45 °C with s/l ratios between 1:50 and 1:250 when using UAE (Figure 1b). MAE performed better when s/l ratios 1:50 or 1:100 were used, regardless of temperature (Figure 1c). Overall, MAC obtained higher yields of gallic acid in comparison with other methods. 

In protocatechuic acid extraction, higher yields when applying all three extraction methods were obtained at extraction temperatures of 75–90 °C. The highest yield of protocatechuic acid was achieved when MAC was used at 90 °C, using s/l ratio 1:500 (Figure 1d). However, UAE was the most suitable method for protocatechuic acid, obtaining twofold higher results in comparison with MAE (Figure 1e). MAE yielded better at 75 °C or higher, regardless of s/l ratio in protocatechuic acid extraction (Figure 1f).

Quercetin-3,4′-diglucoside obtained higher yields when the temperature was set from 75 to 90 °C, regardless of the method. However, a higher yield was observed when MAC was applied using s/l ratios of 1:50 or less (Figure 2a). UAE (Figure 2b) and MAE (Figure 2c) performed better at temperature ranges from 60 to 90 °C in quercetin-3,4′-diglucoside extraction, regardless of the s/l ratio. 

Quercetin-4′-glucoside was extracted in higher amounts using temperature above 75 °C in MAC and UAE (Figure 2d,e), and s/l ratios from 1:25 to 1:100. MAE obtained favorable results at temperature ranges from 60 to 95 °C and s/l ratios from 1:25 to 1:50 (Figure 2f). MAC obtained higher amounts of quercetin-4′-glucoside in general. 

In quercetin-3-glucoside extraction, MAC (Figure 3a) obtained higher yields at 75 or 90 °C, followed by UAE (Figure 3b) using the same temperatures, while MAE performed better at temperatures from 60 °C or higher (Figure 3c) using s/l ratios from 1:25 to 1:50. 

Isorhamnetin-4-glucoside obtained higher values at 75 and 90 °C using s/l ratios from 1:25 to 1:100 in MAC (Figure 3d) and UAE (Figure 3e). As for MAE (Figure 3f), a higher yield was observed when temperature from 45 to 90 °C and s/l ratios from 1:25 to 1:50 were used. 

The results for the antioxidant capacity are available in Supplementary Table S1. The results have shown the influence of temperature in ORAC and FRAP assays when MAC was used, with a low influence of s/l ratios yielding the highest values at 90 °C. On the contrary, DPPH assay results showed the influence of s/l ratio in the extracts; lower values were recorded at all temperatures when the ratio 1:25 was used (Supplementary Table S1). The antioxidant capacity results for UAE indicated highest ORAC values at 45 and 90 °C, regardless of s/l ratio. DPPH results were similar to MAC, while FRAP values suggested higher yields at 75–90 °C regardless of s/l ratio, as shown in Supplementary Table S1. MAE obtained similar results in ORAC and DPPH assays, suggesting higher yields at temperatures from 45 to 75 °C regardless of s/l ratio, but yielding better in the FRAP assay when temperatures of 75 or 90 °C were used.

### 3.2. Adsorption Capacity

#### 3.2.1. Langmuir and Freundlich Isotherms

**Figure 4:**
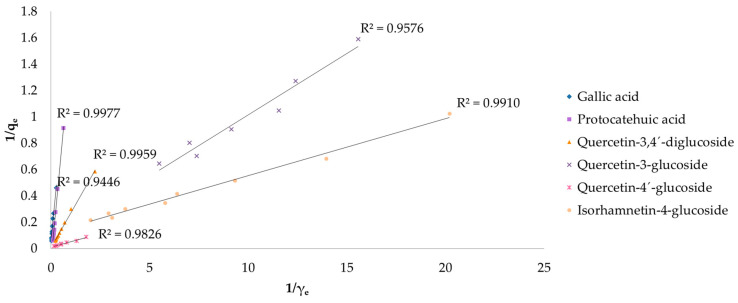
Langmuir isotherms of adsorption of targeted compounds by biochar (R^2^—coefficient of determination, q_e_—amount of adsorbate concentration in the solid phase at equilibrium (mg/g), γ_e_—amount of adsorbate concentration in the liquid phase at equilibrium (mg/L)).

**Figure 5:**
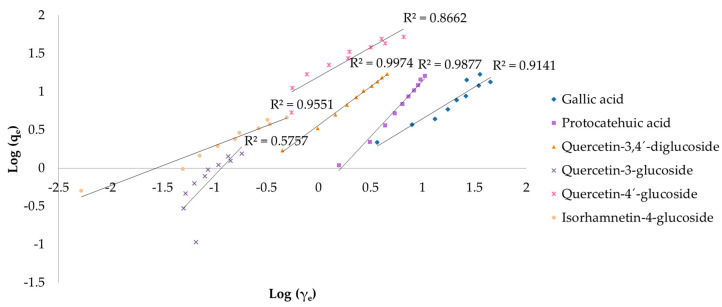
Freundlich isotherms of adsorption of targeted compounds by biochar (R^2^—coefficient of determination, q_e_—amount of adsorbate concentration in the solid phase at equilibrium (mg/g), γ_e_—amount of adsorbate concentration in the liquid phase at equilibrium (mg/L)).

To better understand the adsorption process of targeted compounds by biochar, the results of the first experiment were fitted with the Langmuir and Freundlich isotherm models. Figure 4 shows the Langmuir isotherm models for gallic acid, protocatechuic acid, quercetin-3,4′-diglucoside, quercetin-3-glucoside, quercetin-4′-glucoside, and isorhamnetin-4-glucoside. The R^2^ coefficients ranged from 0.9446 to 0.9977. Freundlich isotherm models are shown in Figure 5, where the R^2^ ranged from 0.5757 to 0.9951. 

The Langmuir model fitted better in gallic acid, quercetin-3-glucoside, and quercetin-4′-glucoside adsorption, while the Freundlich model was more favorable for protocatechuic acid, quercetin-3,4’-diglucoside, and isorhamnetin-4-glucoside (Table 1). As for protocatechuic acid, the compound obtained a higher R^2^ in the Langmuir model, suggesting its suitability. However, the calculated parameters for maximum monolayer adsorption capacity (q_max_) and the affinity constant (K_L_) gave negative results, indicating the inappropriateness of this model for the mentioned compound. Quercetin-3,4’-diglucoside obtained the highest q_max_ value (169 mg/g) followed by quercetin-4′-glucoside which obtained twofold lower (82.0 mg/g). Isorhamnetin-4-glucoside gained the highest affinity constant (K_L_) among other investigated compounds. Using the initial extract concentrations, the R_L_ factor was calculated, resulting in similar values for gallic acid (0.02–0.87) and quercetin-3,4’-diglucoside (0.02–0.90). The highest R^2^ in the Langmuir models was obtained by quercetin-3,4’-diglucoside (0.9959), which also achieved the highest coefficient in the Freundlich models (0.9974). The Freundlich models fitted best for all investigated compounds (R^2^ ≥ 0.9141), except for quercetin-3-glucoside (R^2^ = 0.5757) and quercetin-4′-glucoside (R^2^ = 0.8662), suggesting the unfavorability of this model. The highest adsorption capacity (K_f_) was reached with quercetin-3-glucoside and quercetin-4′-glucoside. The value of 1/n ranged from 0.52 (isorhamnetin-3-glucoside) to 1.43 (quercetin-3-glucoside). 

#### 3.2.2. Biochar Different Dosages in Polyphenolic Adsorption

**Figure 6:**
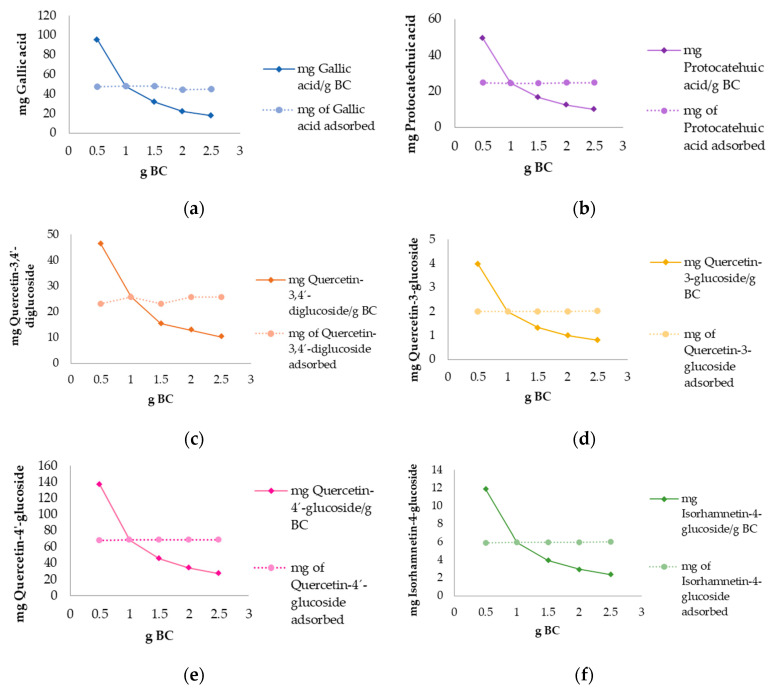
Influence of different biochar (BC) dosages in adsorption of polyphenolic compounds from onion peel extracts in 24 h contact: (**a**) gallic acid; (**b**) protocatechuic acid; (**c**) quercetin-3,4′-diglucoside; (**d**) quercetin-3-glucoside; (**e**) quercetin-4′-glucoside; (**f**) isorhamnetin-4-glucoside.

In Figure 6 the results of the experiment with different biochar dosages using the same concentration of extract (3 g/L) are shown. The results were expressed in mg of targeted compound adsorbed per gram of biochar, and the total amount of adsorbed compound with different biochar dosages applied. The dose of 0.5 g of biochar adsorbed the highest amount of all compounds, with a decreasing trend of adsorption as the amount of biochar increased. As for the total adsorption capacity experiment, there was no influence of biochar dosages on adsorption after reaching equilibrium for all investigated compounds. 

As for gallic acid, the highest amount adsorbed per g of biochar was observed when 0.5 g of BC was applied (95.4 mg gallic acid/g BC), decreasing to 18.1 mg gallic acid/g BC as the biochar dosage increased (Figure 6a). The highest total amount of gallic acid adsorbed was recorded when 0.5, 1, and 1.5 g of biochar were used (47.7–48.0 mg gallic acid) as shown in Figure 6a, slightly decreasing by increasing the biochar dosage. 

The highest amount of protocatechuic acid adsorbed per g of biochar was observed when 0.5 g of BC was applied (49.2 mg protocatechuic acid/g BC), decreasing to 9.84 mg protocatechuic acid/g BC as the biochar dosage increased, as shown in Figure 6b. The total amount of protocatechuic acid adsorbed ranged from 24.5 to 24.6 mg, indicating no influence of biochar dosages (Figure 6b). 

Quercetin-3,4′-diglucoside values in adsorption per g of biochar ranged from 9.84 when 2.5 g of biochar were used to 49.2 mg/g BC at a biochar dosage of 0.5 g (Figure 6c). In the total adsorption experiment, the amount of quercetin-3,4′-diglucoside adsorbed slightly varied by adsorbing 23.2 mg of quercetin-3,4′-diglucoside when 0.5 g of biochar was used, 25.9 mg at 1 g dosage, decreasing to 23.2 mg when 1.5 g of biochar was applied, and finally increasing to 25.9 mg at dosages of 2 and 2.5 g of BC (Figure 6c). 

The amount of adsorbed quercetin-3-glucoside per g of biochar ranged from 18.1 mg when 2.5 g of biochar were used to 95.4 mg in a 0.5 g dosage (Figure 6d), obtaining similar results as other compounds. The total adsorption capacity indicated no influence on quercetin-3-glucoside adsorption when different biochar dosages were applied, obtaining the result of 2.00 mg in all dosages applied (Figure 6d).

Quercetin-4-glucoside was the highest adsorbed compound among others, reaching the value of 137 mg/g BC at a dosage of 0.5 g of biochar (Figure 6e). This compound also showed a trend of decreasing adsorption as the dose of biochar increases. Quercetin-4-glucoside adsorbed values in the total adsorption experiment ranged from 68.3 to 69.3 mg as the biochar dosage increased (Figure 6e). 

The biochar dosage of 0.5 g followed the previous adsorption compounds trend by adsorbing the highest amount of isorhamnetin-4-glucoside (11.9 mg/g BC) and decreasing to 2.41 mg/g BC when 2.5 g of biochar was used (Figure 6f). The total adsorption experiment obtained similar results for all dosages applied, ranging from 5.94 to 6.03 mg of isorhamnetin-4-glucoside adsorbed (Figure 6f). 

## 4. Discussion

In this research, onion peels were used as a source material for polyphenols and biochar from grapevine pruning residues as an adsorbent with the aim to valorize wastes from food and agro-industries. The recovery of bioactive compounds from agro-food wastes should be enforced using eco-friendly, low-cost, and sustainable methods and solvents in order to achieve the principles of green economy [18] and the twelve principles of green chemistry [47]. The aim of green chemistry is to reduce the environmental impact of chemical processes and products [48], leading to the development of alternative solvents. Solvents are widely used as a part of chemical or manufacturing processes [49]. Choosing the appropriate solvent is crucial for dissolution as well as heat and mass transfer [50]. The polarity of targeted compounds could affect the choice of solvents and parameters of the extraction method, respectively [51]. 

According to a group of authors [52], one of the six principles of green extraction includes the use of water as an alternative solvent. Water, as a polar liquid, is very efficient at increasing the solubility of polar or ionic molecules [53]. In this sense, water is a widely used and efficient solvent for extraction of a wide range of compounds [54]. In this work, distilled water was used as a solvent in onion peel extractions in order to reduce the impact on the environment by using organic solvents. 

Lee et al. [55] investigated the influence of solvent on onion peel bioactive compounds yield. Briefly, ethanol, water, and subcritical water were used. Extractions were performed in a water bath using ethanol or water at 60 and 80 °C, respectively. The lyophilized extract mass was higher if water was used (8.31 ± 1.23%) compared to ethanol (4.46 ± 0.22%). However, the total phenolic and flavonoids content was significantly lower in water extractions if compared to other solvents. Nevertheless, a group of authors [56] reported that citrus peels aqueous extracts obtained similar antioxidant capacity results as the methanol extracts, indicating water as an efficient solvent in extractions.

Among all investigated compounds in this study, protocatechuic acid and flavonoids were the most abundant as expected, and their total contents were directly proportional to the antioxidant activity. According to Salehan et al. [57], gallic acid in *Labisia pumila* extract increased when temperature of 50 °C was used, indicating that the extraction proceeded to a certain level and began to decrease due to the compound decomposition. Similar results were obtained in this work, reaching the highest gallic acid yield when using temperatures from 30 to 45 °C. Protocatechuic acid and quercetin-3,4′-diglucoside were extracted in higher amounts at temperature of 75 °C or higher. The amounts of protocatechuic acid at temperatures of 30–45 °C obtained are in accordance with the results obtained by Campone et al. [58], indicating that higher temperatures were more favorable for protocatechuic acid extraction. The yield of quercetin-3,4′-diglucoside and quercetin-3-glucoside were comparable with other results [59,60], both obtaining higher yield when using MAC at 90 °C. Quercetin-4′-glucoside was the richest flavonoid detected, yielding better at 75 °C or higher probably due to the conversion of quercetin-3,4′-diglucoside into quercetin-4′-glucoside, which is further broken into quercetin aglycon as a result of enzymatic hydrolysis of glucosides [61]. Likewise, isorhamnetin-4-glucoside performed similarly to other investigated compounds, yielding better at 75 or 90 °C. According to many authors [62,63,64], the optimum extraction temperature for polyphenols is 60 °C, and higher temperatures are avoided due to potential degradation [65]. However, Xiao et al. [66] obtained valuable results at temperatures 70–110 °C in flavonoid extraction from *Radix astragali*. As the results from this work suggest, there was low or no influence of ratio when MAC and UAE were applied, while a significant difference was observed in MAE application, yielding lower at ratios 1:25 to 1:50, perhaps due to a low amount of material associated with a lower amount of compounds. Lower antioxidant activity and bioactive compounds yield have been observed when MAE was applied. This can be explained as the non-effectiveness of water used as a solvent in MAE of polyphenols when compared to other methods due to reduced dissipation factor and higher dielectric constant associated with water relative to other solvents [67]. Throughout all samples, there was a marked increase in the quantity of investigated compounds, reaching their highest point at ratios 1:100 and 1:250, but then slightly decreasing as the ratio decreased. A similar relationship was also found by Yu et al. [68], probably due to the mass transfer principles [69]. Finally, the obtained results in this research suggest the potential of water as an efficient solvent and the harmlessness of high temperature on antioxidant activity and yield of polyphenolic compound by defining those compounds as heat-labile. 

The method used for polyphenolic extraction from plant materials is important for precise compound quantification and antioxidant activity determination [70]. A growing number of studies are focusing on developing efficient extraction methods that are cost-effective, environmentally friendly, quick, and yield a high amount of bioactive compounds [71]. Different extraction techniques can be applied in polyphenolic compounds recovery. Among green extraction technologies, MAE and UAE are highlighted due to their energy competency, low environmental impact, and relatively high yield [72]. Many papers [29,73] have compared those two methods in bioactive compounds extraction obtaining different results, indicating the importance of the matrix and performing conditions such as solvent, temperature, and ratio. In this work, MAC, UAE, and MAE were performed at the same temperatures from 30–90 °C using the same s/l ratios (1:25, 1:50, 1:100, 1:250 and 1:500) with the aim to compare the suitability of each condition for the specific method, but also to determine which method yielded the best polyphenol extraction from onion peels. Accordingly, antioxidant capacity assays were performed, as well as the identification of individual compounds. The obtained results for ORAC assays indicate higher antioxidant activity of the extracted compounds when performing MAC and UAE at 90 °C and ratios from 1:100 or less. However, slightly lower results were obtained when MAE was used at 60 °C and ratios from 1:50 to 1:250. Celano et al. [15] have investigated the onion peel antioxidant capacity of two different onion varieties. The exhaustive extraction was performed using UAE at 25 °C, s/l ratio 1:20, and aqueous EtOH (70% *v*/*v*) as a solvent. The results ranged from 4.13 to 7.82 µmol TE/g. If compared with the UAE results from this research, by using water, a higher temperature of 5 °C, and a ratio of 1:25 to 1:500, the obtained values were twofold higher. The influence of temperature on FRAP results was more evident compared with other assays. To obtain a higher FRAP potential, extractions at 75 °C or higher should be performed, regardless of ratio and method, albeit MAE obtained comparable results when performed at 60 or 75 °C using ratio 1:25. The obtained results were higher or in range with the literature [74,75]. The DPPH results have shown a significant influence of ratio when MAC and UAE were used. Precisely, the lowest values were recorded when ratio 1:25 was used, regardless of temperature. Nevertheless, the obtained results for all methods applied are in accordance with the research of Bordin Viera et al. [74], in which the radical scavenging activity of purple onion peels extracts against the DPPH radical was investigated, performing extraction on UAE at 25 °C, ratio 1:20 (*m*/*v*), and using different solutions of cereal alcohol as solvent. 

The physicochemical properties of biochar were previously reported, as well as its adsorption potential [41]. In this work, onion peel extract was used as adsorbate due to its abundance in high-value phytochemicals. The adsorption capacity of biochar was determined by fitting the results with Langmuir and Freundlich isotherm models. The Langmuir isotherm theory assumes single-layer coverage of the adsorbate on a homogeneous adsorbent surface with no interactions between adsorbed ions [75], while the Freundlich isotherm assumes multilayer adsorption on heterogeneous surfaces and non-uniform distribution of adsorption heat and affinities to the heterogeneous surface [76]. Among all identified compounds in the extract, gallic acid, protocatechuic acid, quercetin-3,4′-diglucoside, quercetin-3-glucoside, quercetin-4′-glucoside, and isorhamnetin-4-glucoside obtained the best results using these models. All further investigated compounds obtained a high R^2^ coefficient in both models, which is an indicator of the model suitability (the closer the values are to 1, the more suitable the model is), except for protocatechuic acid in the Langmuir model which obtained negative q_max_ and K_L_ values, and quercetin-3-glucoside (R^2^ = 0.5757) and quercetin-4′-glucoside (R^2^ = 0.8662) in the Freundlich model. The negative q_max_ and K_L_ values obtained in the Langmuir model for protocatechuic acid suggest the inadequacy of this model for explaining the adsorption process, although it shows a good linearity compared with other compounds [76]. Furthermore, the negative K_L_ value indicates that at high addition of adsorbent mass, adsorption does not follow Langmuir premises and the adsorption capacity reaches a specific limit by increasing the adsorbent mass at a certain point [77]. The values for maximum monolayer adsorption capacity have shown that the adsorptions of quercetin-3,4′-diglucoside, followed by quercetin-4′-glucoside, were more favorable among other compounds. The K_L_ value, which is related to the energy of adsorption, was significantly higher for isorhamnetin-4-glucoside, indicating a strong interaction between the adsorbent and the adsorbate. The R_L_ coefficient was less than 1 for all investigated compounds, suggesting a favorable adsorption, except for protocatechuic acid which was higher than 1, indicating unfavorable adsorption. The greatest R^2^ was obtained by quercetin-3,4′-diglucoside (R^2^ = 0.9959), perhaps due to the two glucose residues attached at positions 3 and 4′. The mentioned compound gained the highest R^2^ in the Freundlich model as well (R^2^ = 0.9974). The greatest adsorption capacity in the Freundlich model was achieved by quercetin-3-glucoside, which was 20-fold higher if compared to the lowest obtained by gallic acid. The 1/n values of all targeted compounds were in range from 0 to 1, indicating similar adsorption intensity. There are many papers reporting the adsorption potential of biochar obtained from different feedstocks [78,79,80], but according to our knowledge there is no evidence of related work. In our previous work [41], the same biochar was studied using a standard of gallic acid as adsorbate and obtained superior results, probably due to higher initial concentration as reported by Kujawska and Wasag [81] who observed an increased adsorption percentage as the adsorbent dosage increased, as well as the interaction between polyphenols and other extracted compounds such as proteins or carbohydrates, as explained by Jakobek [82]. 

Regarding the biochar dosage experiment, the best adsorption capacity was observed at the lowest biochar dosage (0.5 g) and did not increase by increasing dosage suggesting that the targeted compounds in the solution reached adsorption equilibrium. Identical results were previously reported using the same biochar and polyphenolic standards [41]. Both results are in accordance with other papers [83,84]. This circumstance could have occurred due to the presence of unsaturated adsorption sites during the adsorption process, while the limited adsorption capacity may be due to particle aggregation, resulting from high adsorbent mass [85]. In the research conducted by Chen et al. [86], the influence of biochar dosage on Cd^2+^ adsorption was investigated, among others conditions. The optimum adsorption conditions were obtained using a biochar dosage of 0.4 g which is similar to the results obtained in this work. Regarding the total amount of polyphenols adsorbed, there was no influence on adsorption when different dosages were applied, suggesting the extract concentration played the main role in adsorption.

## 5. Conclusions

In this work, two high-value wastes have been investigated; onion peels as a food waste rich in highly valued phytochemicals, and grapevine pruning residues converted into biochar as a potential adsorbent. Furthermore, three different methods with water as a solvent were applied to obtain onion peel extracts in order to fit with green chemistry principles and lower the impact on the environment. Among MAC, UAE, and MAE, the highest targeted compounds yield and their antioxidant activity were obtained when using MAC or UAE at 75 or 90 °C. The Langmuir and Freundlich isotherms models were used to study the biochar potential in adsorption of polyphenols from onion peel, and the compounds were successfully removed. Onion peel is a food waste material rich in bioactive compounds which could be implemented in different industries due to their wide benefits on human health, and biochar from grapevine pruning residues shows a high potential in adsorption of those compounds. Biochar extraction of polyphenols from onion peels results in a win–win situation for biomass valorization considering the use of wasted material containing high-value compounds, while using biochar obtained from the grapevine pruning residues which is a waste product in viticulture. Further research should be performed for the recovery of the adsorbed compounds. 

## Figures and Tables

**Table 1:** Langmuir and Freundlich model parameters for targeted onion peel polyphenolic compounds adsorbed by grapevine pruning residues biochar.

Type of Isotherm	Parameters	GA	PA	Q-3,4′-d	Q-3-g	Q-4′-g	I-4-g
Langmuir	q_max_ (mg/g)	20.8	−10.4	169	12.1	82.0	7.22
	K_L_ (L/mg)	0.03	−0.06	0.02	0.89	0.30	3.51
	R_L_	0.02–0.87	0.01–1.43	0.02–0.90	0.10–0.18	0.05–0.40	0.05–0.27
	R^2^	0.9446	0.9977	0.9959	0.9576	0.9826	0.9869
Freundlich	K_f_ (mg/g) × (L/g)^n^	0.65	1.69	3.63	21.6	15.6	6.53
	1/n	0.83	0.68	1.01	1.43	0.76	0.52
	R^2^	0.9141	0.9877	0.9974	0.5757	0.8662	0.9951

GA—gallic acid; PA—protocatechuic acid; Q-3,4′-d—quercetin-3,4′-diglucoside; Q-3-g—quercetin-3-glucoside; Q-4′-g—quercetin-4′-glucoside; I-4-g—isorhamnetin-4-glucoside.

## Data Availability

Not applicable.

## Supplementary Materials

The following supporting information can be downloaded at: https://www.mdpi.com/article/10.3390/antiox12091697/s1, Table S1. Onion peels antioxidant capacity using MAC, UAE and MAE at different extraction conditions.
