# Supplementary material for: From Waste to Green: Water-Based Extraction of Polyphenols from Onion Peel and Their Adsorption on Biochar from Grapevine Pruning Residues
Source: Antioxidants (Basel). 2023 Aug 31;12(9):1697. doi: 10.3390/antiox12091697 (PMC10525769; doi:10.3390/antiox12091697)
Supplement: Supplementary file 1 [file antioxidants-12-01697-s001.zip › antioxidants-2542697-supplementary.pdf]

Supplementary Table S1. Onion peels antioxidant capacity using MAC, UAE and MAE at different extraction conditions.

Assay	Condition		Method			Method x temperature x ratio (p – value)
	Temperature (°C)	Solid/liquid ratio	MAC	UAE	MAE	
ORAC (μmol TE/g)	30	1:25	15.0 ± 0.4 g-i	12.8 ± 0.3 e	2.22 ± 0.47 i	***
		1:50	15.8 ± 0.3 f-i	15.4 ± 0.9 c-e	3.16 ± 0.28 i	
		1:100	15.7 ± 0.0 f-i	18.8 ± 0.7 a-e	14.4 ± 0.4 gh	
		1:250	17.0 ± 0.7 d-i	16.9 ± 0.3 b-e	16.6 ± 0.9 d-h	
		1:500	19.1 ± 0.7 c-g	15.9 ± 0.4 b-e	13.6 ± 0.7 h	
	45	1:25	10.3 ± 0.6 j	26.3 ± 9.5 ab	19.0 ± 0.2 b-e	
		1:50	13.8 ± 0.2 ij	20.0 ± 0.5 a-e	18.1 ± 0.8 c-g	
		1:100	19.0 ± 0.4 c-h	23.2 ± 0.6 a-e	22.0 ± 1.6 a-c	
		1:250	16.3 ± 0.5 e-i	24.0 ± 0.7 a-d	16.7 ± 0.6 d-h	
		1:500	14.0 ± 0.6 ij	22.8 ± 0.1 a-e	14.6 ± 1.6 f-h	
	60	1:25	14.8 ± 0.2 hi	17.8 ± 0.4 a-e	19.3 ± 0.2 b-e	
		1:50	16.7 ± 0.3 e-i	18.8 ± 0.1 a-e	22.6 ± 0.6 ab	
		1:100	20.5 ± 0.8 c-e	22.4 ± 0.6 a-e	23.8 ± 0.1 a	
		1:250	21.7 ± 0.0 bc	16.7 ± 0.0 b-e	21.5 ± 0.4 a-c	
		1:500	19.7 ± 1.4 c-f	13.6 ± 0.2 de	17.2 ± 1.5 d-h	
	75	1:25	18.4 ± 0.9 c-h	17.0 ± 0.5 b-e	19.6 ± 0.7 b-e	
		1:50	19.2 ± 0.6 c-g	18.4 ± 0.6 a-e	18.0 ± 0.5 c-g	
		1:100	21.3 ± 0.6 b-d	17.4 ± 0.1 a-e	22.4 ± 1.1 ab	
		1:250	17.7 ± 0.3 c-i	17.3 ± 0.1 a-e	15.8 ± 0.5 e-h	
		1:500	18.4 ± 0.0 c-h	17.0 ± 0.9 b-e	15.9 ± 0.4 d-h	
	90	1:25	17.7 ± 0.2 c-i	18.8 ± 0.2 a-e	20.1 ± 0.3 a-d	
		1:50	19.7 ± 2.1 c-f	18.4 ± 0.5 a-e	18.7 ± 1.3 b-f	

		1:100	27.3 ± 1.1 a	27.9 ± 0.4 a	18.5 ± 0.3 b-g	
		1:250	25.5 ± 1.1 ab	24.6 ± 0.0 a-c	18.0 ± 0.6 c-g	
		1:500	24.9 ± 1.2 ab	23.8 ± 0.2 a-d	15.9 ± 0.1 e-h	
DPPH (µmol TE/g)	30	1:25	24.9 ± 0.9 h	27.4 ± 1.8 i	18.1 ± 0.7 h	***
		1:50	39.6 ± 4.5 c-h	50.0 ± 3.1 c-f	40.3 ± 6.1 c-g	
		1:100	44.9 ± 2.2 b-g	51.5 ± 1.7 c-f	49.4 ± 1.2 b-e	
		1:250	58.9 ± 0.4 ab	46.0 ± 0.6 e-h	46.9 ± 1.9 b-f	
		1:500	39.2 ± 3.8 c-h	41.2 ± 2.5 f-i	37.8 ± .3.1 d-g	
	45	1:25	30.9 ± 4.9 e-h	30.9 ± 1.4 hi	35.5 ± 1.3 e-g	
		1:50	42.2 ± 3.8 b-h	56.7 ± 2.6 a-f	45.3 ± 2.7 b-f	
		1:100	42.1 ± 1.6 b-h	61.3 ± 6.5 a-e	57.8 ± 1.4 ab	
		1:250	41.3 ± 2.3 b-h	54.8 ± 0.6 b-f	52.6 ± 4.5 a-c	
		1:500	49.3 ± 7.3 a-e	47.6 ± 3.1 d-g	40.8 ± 1.8 c-g	
	60	1:25	26.4 ± 0.8 gh	32.5 ± 1.8 g-i	34.1 ± 0.8 fg	
		1:50	46.9 ± 1.5 a-f	55.4 ± 1.3 b-f	51.9 ± 3.4 a-d	
		1:100	51.8 ± 2.5 a-c	72.0 ± 3.7 a	53.9 ± 4.0 a-c	
		1:250	48.1 ± 4.6 a-e	60.1 ± 0.3 a-e	53.1 ± 2.1 a-c	
		1:500	41.0 ± 2.9 b-h	52.5 ± 4.9 b-f	41.8 ± 1.8 c-g	
	75	1:25	29.5 ± 2.9 f-h	31.1 ± 0.0 hi	29.5 ± 0.3 gh	
		1:50	49.5 ± 1.6 a-e	61.7 ± 0.4 a-e	52.5 ± 0.9 a-c	
		1:100	57.1 ± 3.7 a-c	60.9 ± 2.1 a-e	64.2 ± 3.7 a	
		1:250	40.2 ± 4.9 c-h	63.2 ± 3.7 a-d	51.7 ± 3.7 a-d	
		1:500	39.1 ± 0.9 c-h	54.5 ± 0.7 b-f	36.1 ± 1.1 e-g	
	90	1:25	32.4 ± 1.0 d-h	32.5 ± 1.0 g-i	34.1 ± 3.4 fg	
		1:50	50.0 ± 1.0 a-d	63.0 ± 7.8 a-d	39.2 ± 1.0 c-g	

		1:100	47.4 ± 6.5 a-f	61.0 ± 1.6 a-e	41.7 ± 2.4 c-g	
		1:250	64.1 ± 2.6 a	68.7 ± 2.3 ab	46.2 ± 1.4 b-f	
		1:500	52.1 ± 3 a-c	64.3 ± 3.6 a-d	35.6 ± 2.9 e-g	
FRAP (µmol TE/g)		1:25	78.6 ± 7.5 g-j	74.6 ± 0.8 l	20.0 ± 1.2 k	***
		1:50	60.3 ± 2.3 k	95.9 ± 1.0 g-j	30.9 ± 1.5 k	
	30	1:100	64.5 ± 3.1 jk	88.6 ± 1.8 i-k	57.9 ± 2.7 j	
		1:250	71.2 ± 3.0 i-k	98.0 ± 1.0 g-i	61.7 ± 2.0 ij	
		1:500	84.1 ± 0.3 e-i	75.1 ± 0.5 l	55.4 ± 4.0 j	
		1:25	85.0 ± 4.6 e-i	89.7 ± 2.4 i-k	90.5 ± 2.4 c-e	
		1:50	87.4 ± 1.2 d-i	84.4 ± 0.8 j-l	79.5 ± 2.4 e-g	
		1:100	87.3 ± 1.1 d-i	72.6 ± 5.4 l	77.0 ± 1.3 f-h	
	45	1:250	95.7 ± 3.7 c-g	97.1 ± 2.0 g-i	74.4 ± 3.4 g-i	
		1:500	88.2 ± 0.9 d-i	88.9 ± 1.7 i-k	58.5 ± 0.5 j	
		1:25	96.6 ± 0.2 c-g	108 ± 4 fg	101 ± 3 bc	
		1:50	81.1 ± 1.3 f-j	93.9 ± 1.5 h-j	87.5 ± 1.8 d-f	
	60	1:100	88.1 ± 4.5 d-i	90.8 ± 2.5 i-k	84.3 ± 3.5 d-g	
		1:250	90.3 ± 1.2 c-h	78.6 ± 1.6 kl	75.6 ± 1.0 f-h	
		1:500	76.0 ± 1.6 h-k	74.6 ± 0.7 l	66.0 ± 5.0 h-j	
		1:25	79.5 ± 1.9 f-j	112 ± 3 ef	121 ± 4 a	
		1:50	100 ± 2 c-e	107 ± 1 fg	82.5 ± 1.4 d-g	
	75	1:100	108 ± 1 bc	104 ± 1 f-h	84.3 ± 0.9 d-g	
		1:250	103 ± 1 cd	98.2 ± 0.5 g-i	87.1 ± 1.2 d-g	
		1:500	105 ± 1 b-d	95.5 ± 0.8 g-j	79.4 ± 1.4 e-g	
	90	1:25	130 ± 3 a	133 ± 2 cd	120 ± 2 a	
		1:50	121 ± 9 ab	121 ± 2 de	113 ± 2 ab	

	1:100	129 ± 2 a	139 ± 5 bc	102 ± 2 bc
	1:250	139 ± 4 a	152 ± 3 a	95.1 ± 0.7 cd
	1:500	134 ± 1 a	146 ± 1 ab	76.9 ± 0.3 f-h

MAC – maceration; UAE – ultrasound-assisted extraction; MAE – microwave-assisted extraction; TE – Trolox equivalents; *** – p-value ≤ 0.001. Small letters represent different homogenous groups using Tukey's post-hoc test.
